# Supplementary material for: Features of membrane protein sequence direct post-translational insertion
Source: Nat Commun. 2024 Nov 25;15:10198. doi: 10.1038/s41467-024-54575-6 (PMC11589881; doi:10.1038/s41467-024-54575-6)
Supplement: Supplementary file 1 — Supplementary Information [file 41467_2024_54575_MOESM1_ESM.pdf]

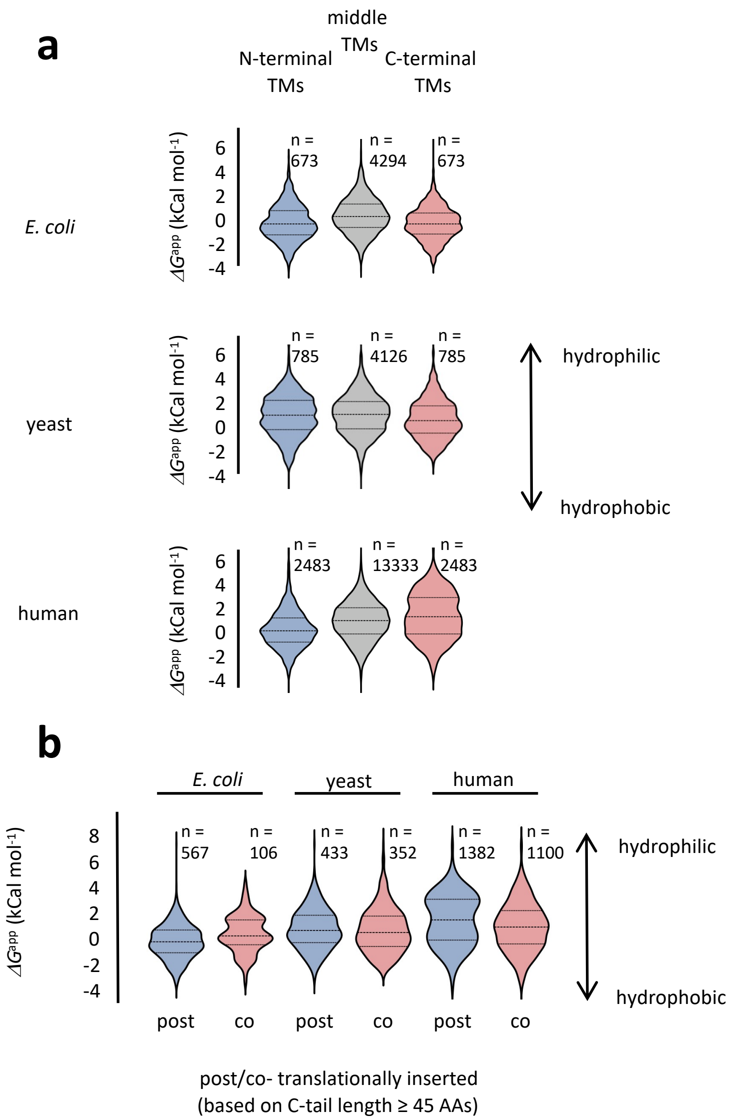

Supplementary Fig. 1. Hydrophobicities of TMs placed in different positions in multispanning proteins.

The hydrophobicities of all TMs were calculated according to the biological hydrophobicity scale (Hessa et al., 2007), which predicts the ΔG^{app} for membrane insertion. Lower values indicate higher hydrophobicity. **(a)** Hydrophobicities of TMs placed at the terminal positions, either N- or C-terminal, compared to middle TMs. The cTMs display similar hydrophobicities. **(b)** The Hydrophobicities of cTMs that precede a C-tail of ≥ 45 amino acids, which can theoretically get inserted cotranslationally, are similar to cTMs with short C-tails that necessitate posttranslational insertion.

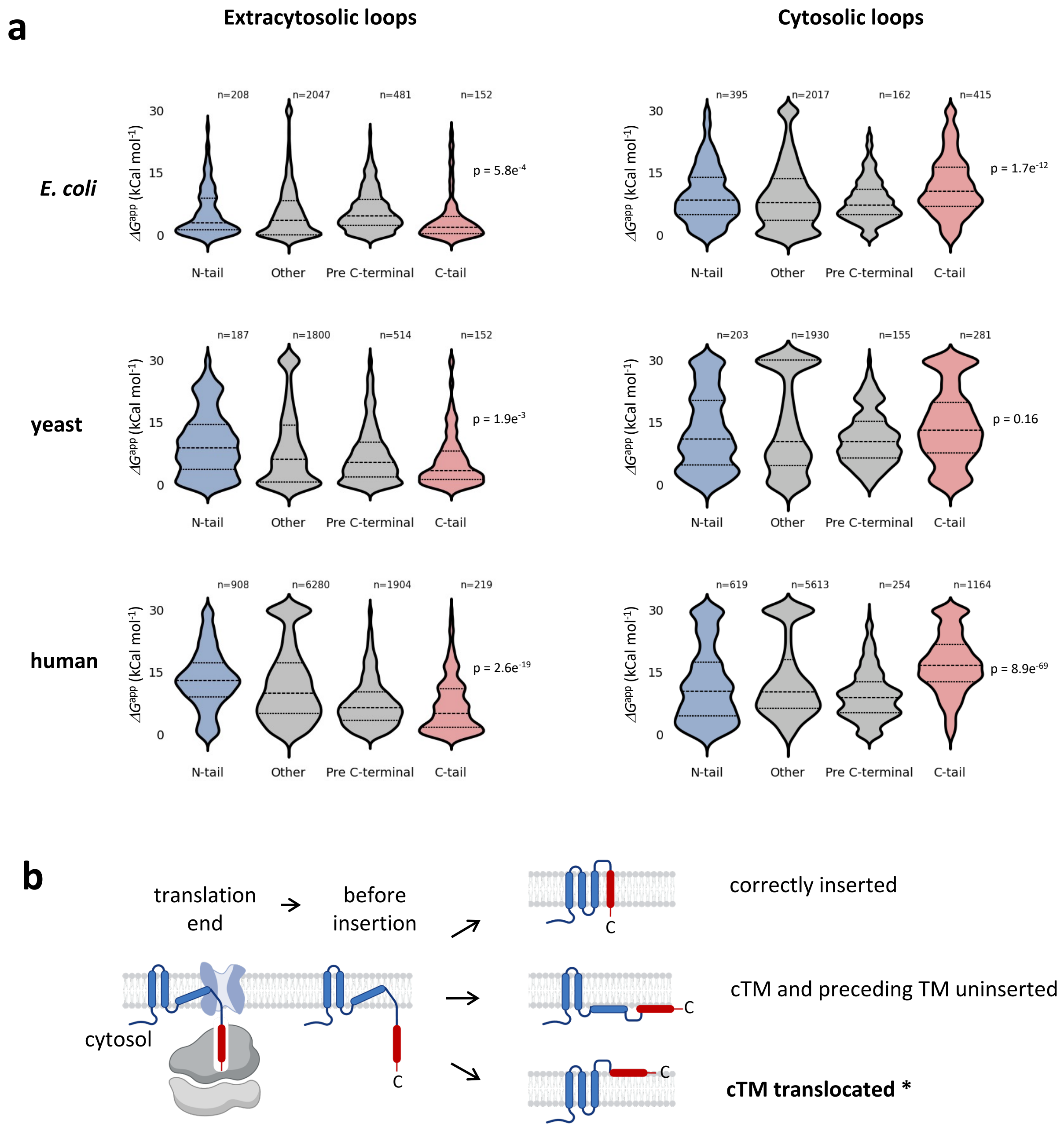

Supplementary Fig. 2. Hydrophilicities of loops and tails in different positions along the protein and different membrane sides. (a) C-tails possess significantly different hydrophilicities compared to other loops or tails found on the same side of the membrane. 'Pre C-terminal' refers to the loop preceding the cTM. The indicated P-values are for comparison between the C-tail and 'Other' loops, two-sided Mann-Whitney test. Only loops/tails shorter than 45 residues were taken for analysis. **(b)** Possible topologies after correct or erroneous posttranslational cTM insertion in C_{cyt} proteins. * marks a potential insertion error, in which the cTM, along with the C-tail, is translocated to the extracytosolic side. This error might be alleviated by a hydrophilic C-tail anchoring the C-tail to the cytosol. Created in BioRender. Kalinin, I. (2023) BioRender.com/s44q492.

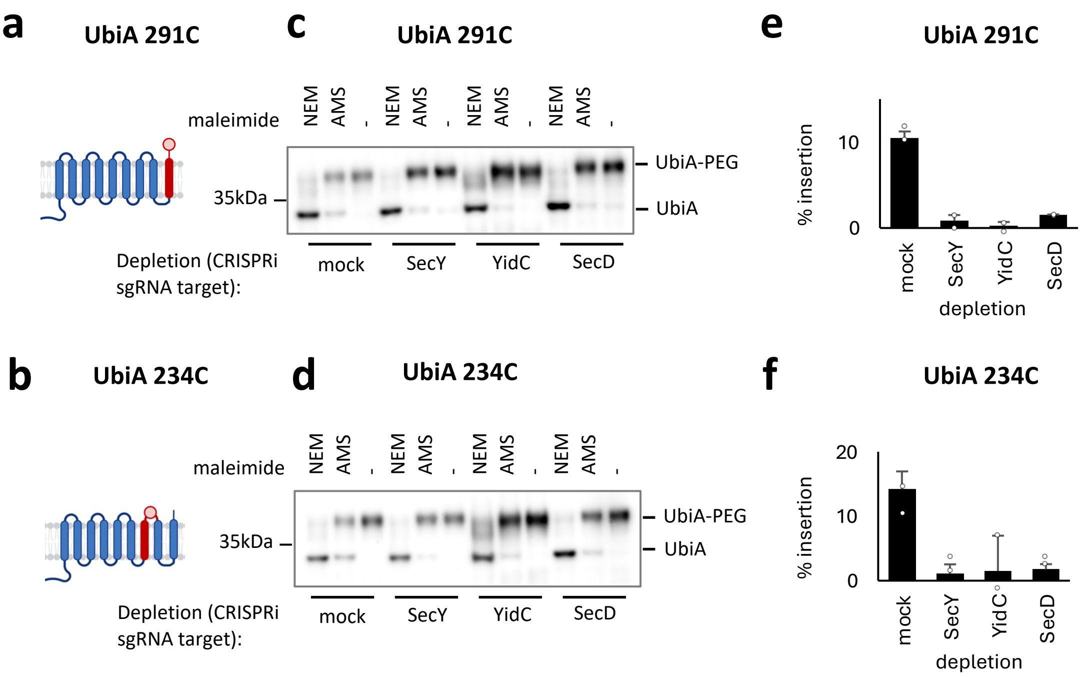

Supplementary Fig. 3. Depleting insertion factors affects the insertion of multiple TMs of the full-length multi-spanning protein UbiA.
(a,b) A Cys was introduced in two different positions in UbiA to analyze the insertion of distinct regions of the protein; UbiA-291C is used to probe cTM insertion (a), and UbiA-234C probes TM7 and TM8 (b). Created in BioRender. Kalinin, I. (2023) BioRender.com/s22f543. **(c,d)** Depletion of SecY , YidC and SecD disrupts the insertion of both tested loops of the full-length UbiA. Notably, expression of the CRISPRi system reduced the baseline level of insertion, even under mock sgRNA targeting none of the insertion factors, compared to Fig. 2c. The reason for this reduction is unclear. **(e,f)** Quantitative analysis of two biological replicates, shown means and SEM.

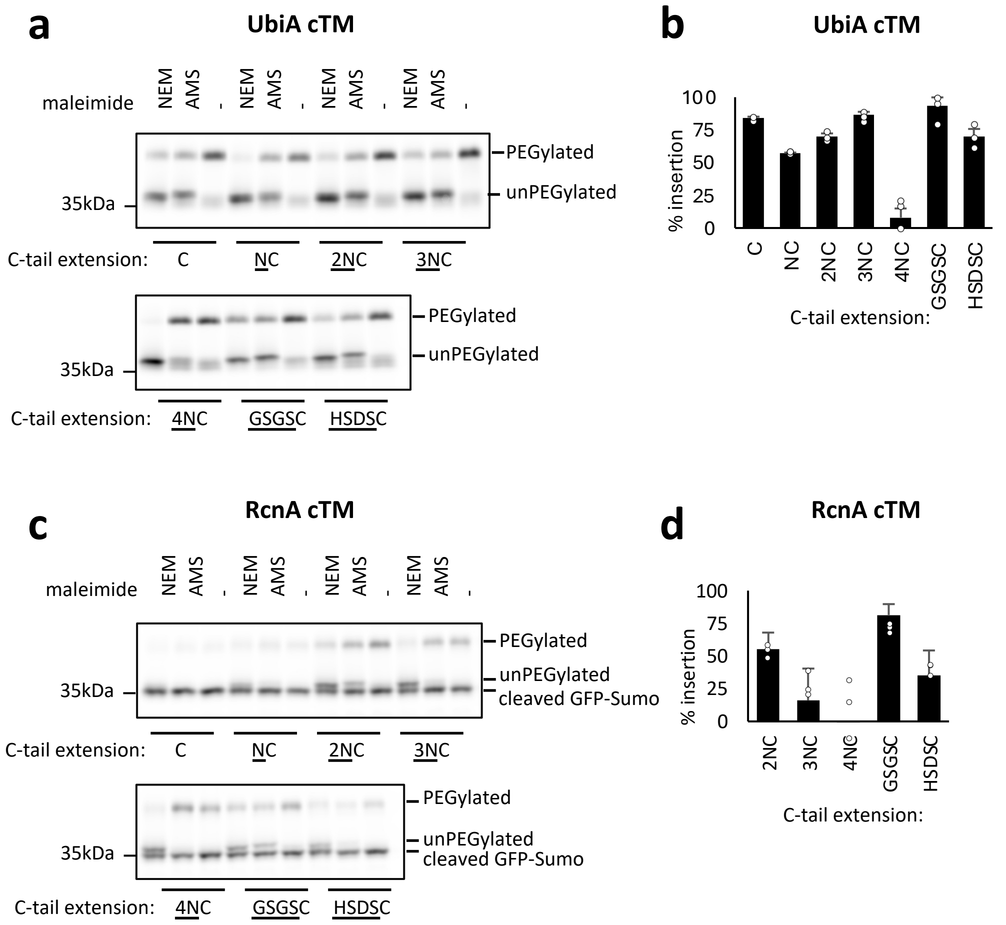

Supplementary Fig. 4. The preference for short and hydrophobic C-terminal tails is largely retained in chimeric His$_6$-GFP-Sumo-cTM constructs.
(a,c) The *in vivo* insertion of His$_6$-GFP-Sumo-cTM constructs, harboring the cTM of UbiA (a) or RcnA (c), was assessed by their reaction with AMS and NEM, which blocks PEGylation, similar to Fig. 2c,d. The migration of the proteins on SDS-PAGE was visualized by in-gel fluorescence. Note that RcnA-cTM constructs display a significant band consistent with cleaved GFP-Sumo harboring no cTM nor Cys, which does not get PEGylated. This band represents the major protein form for the C and NC extensions, and therefore their topologies cannot be determined and were excluded from further analysis. The gradual increase in hydrophilicity disrupts the insertion of the cTM chimeric protein in a manner similar to the full-length proteins. **(b,d)** Quantitative analysis of three biological repeats is shown for UbiA-cTM and RcnA-cTM. Shown are means ± SEM.

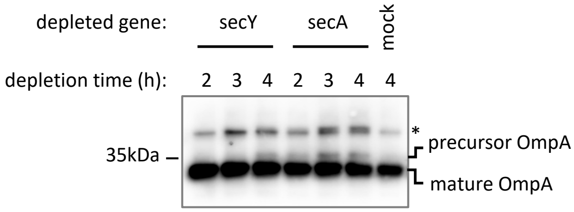

Supplementary Fig. 5. Four hours of CRISPRi depletion is sufficient to inactivate SecY and SecA. *E. coli* cells were grown under CRISPRi depletion conditions that inhibit the transcription of the indicated genes for 2-4 hours, and the OmpA protein was detected by Western blotting. The inactivation of SecY and SecA after four hours of depletion, which is the duration of depletion in our experiments, is indicated by the appearance of an OmpA precursor due to secretion impairment. Note that since OmpA is a stable protein, most of the OmpA in these samples has been synthesized and matured before the depletion, and hence the mature form is dominant.

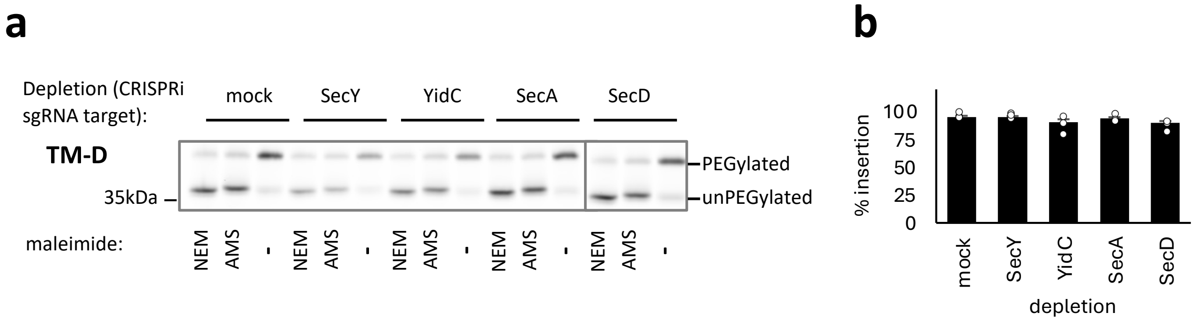

Supplementary Fig. 6. Depletion of Sec components or YidC does not impair the insertion of TM-D. Panels **a** and **b** are similar to Fig. 3 b and c, respectively, except that the chimeric His_6-GFP-Sumo-TM construct harbored the TM-D transmembrane helix.

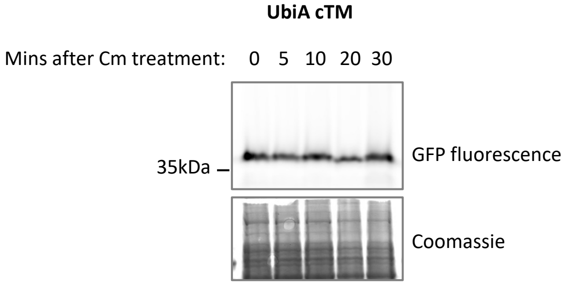

Supplementary Fig. 7. Translation shutdown by Chloramphenicol (Cm). The fluorescence intensity of His6-GFP-Sumo-UbiA-cTM does not increase following addition of Cm to the culture medium, suggesting that protein synthesis is effectively halted.

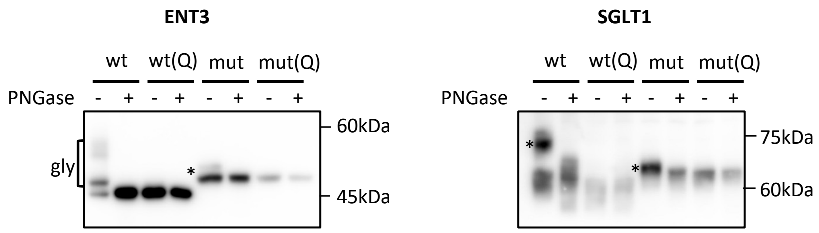

Supplementary Fig. 8. Elimination of ENT3 and SGLT1 endogenous glycosylation sites. The endogenous glycosylation sites of ENT3 and SGLT1 were eliminated by mutating aspargine 84 of ENT3 and aspargine 248 of SGLT1 to glutamine (Q). HEK293T cells were transiently transfected with wildtype (wt), nonstop mutants (mut) or their glycosylation mutants (wt(Q), and mut(Q), respectively) and analyzed with immunoblotting with an anti-HA antibody. Elimination of the endogenous glycosylation site reduced the apparent size of the proteins similarly to treatment with PNGase F. Glycosylated protein bands are indicated by [or *. Note that wt-SGLT1 displays two bands of slightly different gel migration. Representative blots of 3 biological repeats are shown.

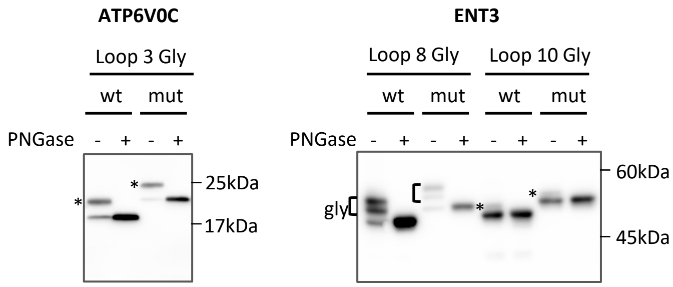

Supplementary Fig. 9. The extracytosolic loops preceding the extended C-terminals of ATP6V0C and ENT3 mutants are correctly inserted. The endogenous glycosylation site of ENT3 and its nonstop mutants was eliminated by mutation (Q, see Fig. S8) and an opsin glycosylation tag was added to extracytosolic loop 3 of ATP6V0C (Loop 3 Gly) and extracytosolic loops 8 and 10 of ENT3 (Loop 8 Gly and Loop 10 Gly, respectively). An increase in the apparent size of the opsin-tagged proteins that is eliminated upon PNGase F treatment suggests that the tagged loops face the ER lumen, as expected if they are correctly inserted. Glycosylated protein bands are indicated by [or *. Representative blots of 3 biological repeats are shown.

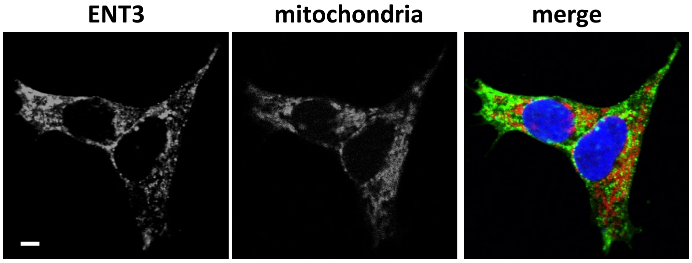

Supplementary Fig. 10. ENT3 is not localized to mitochondria in HEK293 cells.
HEK293 cells transfected with HA-ENT3 were treated with MitoTracker® Deep Red FM and then immunostained with anti-HA antibodies. In the merged image, ENT3 is shown in green, the mitochondria marker is shown in red and DAPY nuclear staining is shown in blue. Scale bar, 5 μm.

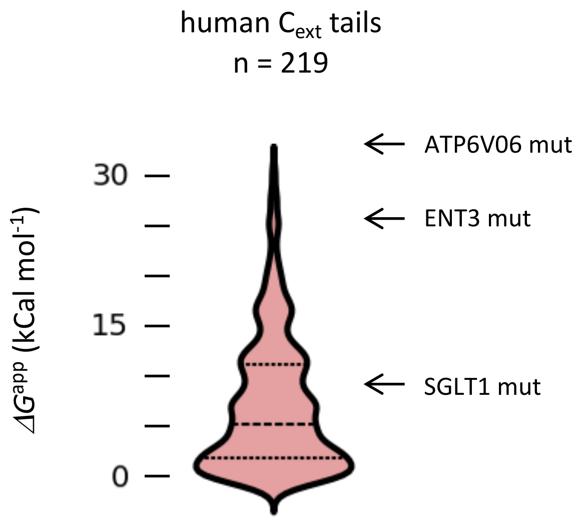

Supplementary Fig. 11. The distribution of C-tail hydrophilicities in natural human proteins compared to the nonstop mutants of SGLT1, ENT3 and ATP6V06

Supplementary table 1. Primers used in this study

Primer name	Sequence (5' -> 3')	application
**RcnA_C F**	TGCGATGCTAAtgatacagattaaatcagaacgcagaagc	Full length RcnA series
**RcnA_C R**	gtatcaTTAGCATCGCATTATGCCCATGAAGCC	Full length RcnA series
**RcnA_NC F**	GCGAAATTGCTAAtgatacagattaaatcagaacgca	Full length RcnA series
**RcnA_NC R**	TTAGCAATTTCGCATTATGCCCATGAAGCC	Full length RcnA series
**RcnA_2NC F**	GCGAAATAACTGCTAAtgatacagattaaatcagaacgca	Full length RcnA series
**RcnA_2NC R**	TTAGCAGTTATTTCGCATTATGCCCATGAAGCC	Full length RcnA series
**RcnA_3NC F**	GCGAAATAACAATTGCTAAtgatacagattaaatcagaacgca	Full length RcnA series
**RcnA_3NC R**	TTAGCAATTGTTATTTCGCATTATGCCCATGAAGCC	Full length RcnA series
**RcnA_4NC F**	GCGAAATAACAATAACTGCTAAtgatacagattaaatcagaacgca	Full length RcnA series
**RcnA_4NC R**	TTAGCAGTTATTGTTATTTCGCATTATGCCCATGAAGCC	Full length RcnA series
**RcnA_HSDS F**	CATAATGCGAcatagcgattcttgcTAAtgatacagattaaatcagaac	Full length RcnA series
**RcnA_HSDS R**	gtatcaTTAgcaagaatcgctatgTCGCATTATGCCCATGAAG	Full length RcnA series
**RcnA_GSGS F**	CATAATGCGAggtagcggctcttgcTAAtgatacagattaaatcagaac	Full length RcnA series
**RcnA_GSGS R**	gtatcaTTAgcaagagccgctaccTCGCATTATGCCCATGAAG	Full length RcnA series
**RcnA_opsin F**	CgttcagcaacaaaaccggcTGCTAAtgatacagattaaatcagaac	Full length RcnA series
**RcnA_opsin R**	gcacatagaagttcgggccTCGCATTATGCCCATGAAG	Full length RcnA series
**UbiA_C F**	CATTTCtgcTGATAGCCATCTTCGTTCACGTTTG	Full length UbiA series
**Ubi_C R**	CTATCAgcaGAAATGCCAGTAACTCATTGCCAG	Full length UbiA series
**UbiA _NC F**	CATTTCaactgcTGATAGCCATCTTCGTTCACGTTTGC	Full length UbiA series
**UbiA_NC R**	TCAgcagttGAAATGCCAGTAACTCATTGCCAGCCC	Full length UbiA series
**UbiA_2NC F**	CATTTCaacaattgcTGATAGCCATCTTCGTTCACGTTTGC	Full length UbiA series
**UbiA_2NC R**	TCAgcaattgttGAAATGCCAGTAACTCATTGCCAGCCC	Full length UbiA series
**UbiA_3NC F**	CATTTCaacaataactgcTGATAGCCATCTTCGTTCACGTTTG C	Full length UbiA series
**UbiA_3NC R**	TCAgcagttattgttGAAATGCCAGTAACTCATTGCCAGCCC	Full length UbiA series
**UbiA_4NC F**	taacaattgcTGATAGCCATCTTCGTTCAC	Full length UbiA series
**UbiA_4NC R**	CAgcaattgttattgttGAAATGCCAGTAACTCATTGC	Full length UbiA series
**UbiA_HSDS F**	CTGGCATTTCcatagcgattcttgcTGATAGCCATCTTCG	Full length UbiA series
**UbiA_HSDS R**	CTATCAgcaagaatcgctatgGAAATGCCAGTAACTCATTGC	Full length UbiA series
**UbiA_GSGS F**	CggtagcggctcttgcTGATAGCCATCTTCGTTCACG	Full length UbiA series
**UbiA_GSGS R**	CTATCAgcaagagccgctaccGAAATGCCAGTAACTCATTGC	Full length UbiA series
**UbiA_opsin F**	CgttcagcaacaaaaccggctgcTGATAGCCATCTTCG	Full length UbiA series
**UbiA_opsin R**	gcacatagaagttcgggccGAAATGCCAGTAACTCATTGC	Full length UbiA series
**UbiA_234C F**	GTTAAATtGCTTAGGCTGGGGATATTACTGGTC	Internal Cys of UbiA
**UbiA_234C F**	CTAAGCaATTTAACTCACCGATAATCGCCATCAGC	Internal Cys of UbiA
**Sumo_R**	GACTTTTTAGAACCGCCACCTCCTTTG	sfGFP_sumo_cTM constructs
**TMUbiA_F**	TTTAAAGCATTTATGAATAATAACTATGTTGGTCTGGTACT GTTTTTAG	sfGFP_sumo_cTM constructs
**TMRcnA_F**	TATTTTTCCAGTCTGTTGATTGGCTTAGTCGGTG	sfGFP_sumo_cTM constructs
**Sumo_F**	CAAAGGAGGTGGCGGTTCTAAAAAGTC	sfGFP_sumo_cTM constructs
**TMUbiA_R**	CATAGTTATTATTCATAAATGCTTTAAAGCTTTTCTTGCTG CCACCCCCG	sfGFP_sumo_cTM constructs
**TMRcnA_R**	CCGACTAAGCCAATCAACAGACTGGAAAAATAGCTTTTC TTGCTGCCACCCCCG	sfGFP_sumo_cTM constructs
**ENT_NtQ_F**	ctccgcCaGtcctccagcccagccacc	abolishing native glycosylation site
**ENT_NtQ_R**	gaggaCtGgcggagtttgaacatccagtactcc	abolishing native glycosylation site

Primer name	Sequence (5’ -> 3’)	application
**SGLT_NtQ_F**	gatggcCaGaccacctttcaggaaaaatgctacactc	abolishing native glycosylation site
**SGLT_NtQ_R**	gtggtCtGgccatcagacactatggttggaatgg	abolishing native glycosylation site
**ATP_NFT_F**	tCACATACGCACGGGGCCG	c-tail glycosylation site
**ATP_NFT_R**	agttCTGTTCGTTCTGGAATGAGGAGGGG	c-tail glycosylation site
**ENT_NFT_F**	actttaCcGGGGGCCATGGAGGAAAG	c-tail glycosylation site
**ENT_NFT_R**	TGCACTCTCTTCTCATCTTCAAAGGCTC	c-tail glycosylation site
**SGLT_NFT_F**	AgttTGAGTAAGAGTCCAGCCATGGTAAATCTAC	c-tail glycosylation site
**SGLT_NFT_F**	tCaccAGTCTCGTCCTGTGGTGTTGAtagag	c-tail glycosylation site
**ATP_L3gly F**	GTCCCCTTTTCCAACAAGACCGTGGACtccctgaatgacgacatcagcctc	Internal loop opsin site
**ATP_L3gly R**	GTAAAAGTTGGGTCCCTCGGTTCCGTTCATgttggcgatgaggactgccac	Internal loop opsin site
**ENT_L8gly F**	GTCCCCTTTTCCAACAAGACCGTGGACggctcgggctcactgtggac	Internal loop opsin site
**ENT_L8gly R**	GTAAAAGTTGGGTCCCTCGGTTCCGTTCATcttgttgagggactcgatgttggtg	Internal loop opsin site
**ENT_L10gly F**	GTCCCCTTTTCCAACAAGACCGTGGACgtccacctgaagactgtggtcttc	Internal loop opsin site
**ENT_L10gly R**	CGTAAAAGTTGGGTCCCTCGGTTCCGTTCATgcggggctggtagttacagag	Internal loop opsin site
