# Supplementary material for: Features of membrane protein sequence direct post-translational insertion
Source: Nat Commun. 2024 Nov 25;15:10198. doi: 10.1038/s41467-024-54575-6 (PMC11589881; doi:10.1038/s41467-024-54575-6)
Supplement: Supplementary file 2 — Description of Additional Supplementary Files [file 41467_2024_54575_MOESM2_ESM.pdf]

**File name: Supplementary Data 1**
Description: Topology predictions of the membrane proteomes of *E. coli*, yeast and human.
