# Supplementary material for: Features of membrane protein sequence direct post-translational insertion
Source: Nat Commun. 2024 Nov 25;15:10198. doi: 10.1038/s41467-024-54575-6 (PMC11589881; doi:10.1038/s41467-024-54575-6)

Figure 3c UbiA-cTM

Repeat 1

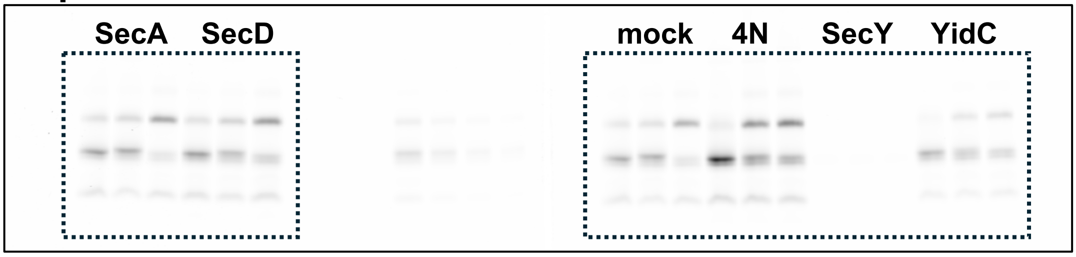

Repeat 2

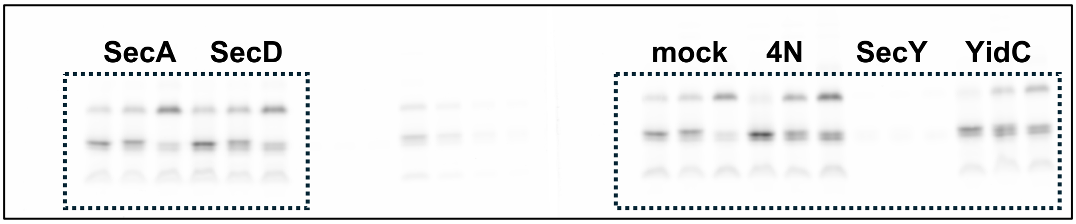

Repeat 3

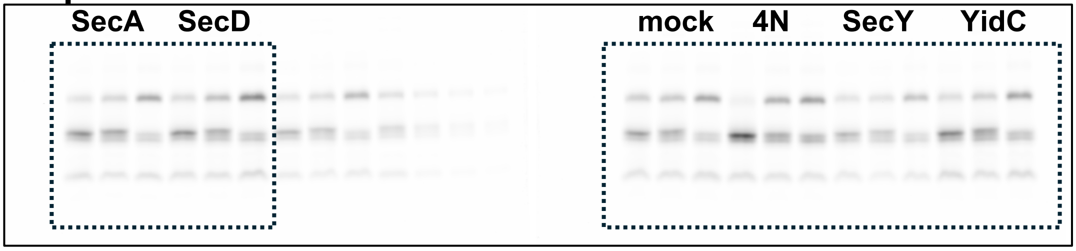

Figure 3c RcnA-cTM

Repeat 1

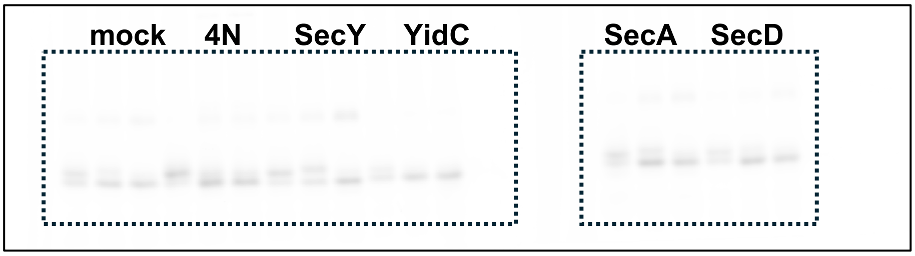

Repeat 2

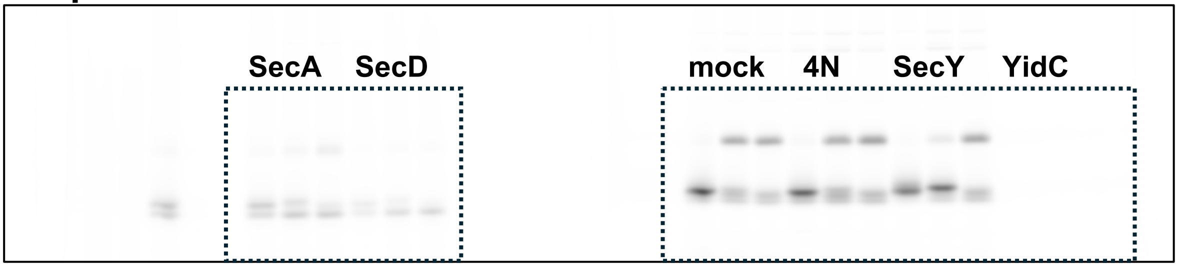

Repeat 3

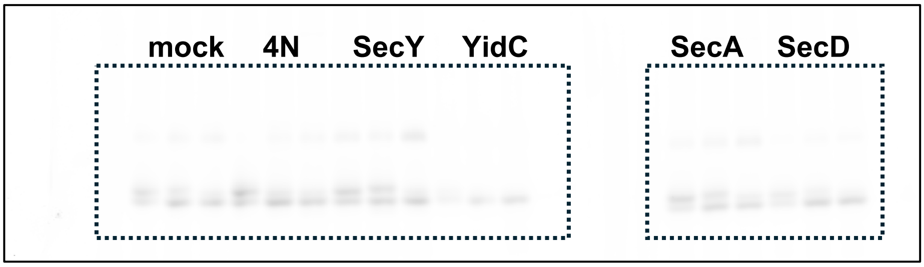

Figure 3d (panel no. 1, 2, 4),

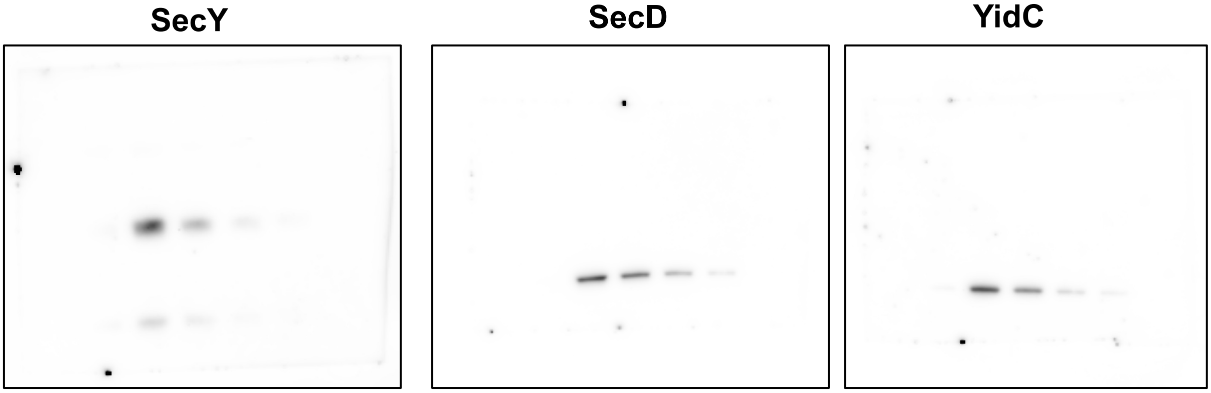

Figure 3e (panel no. 1-4)

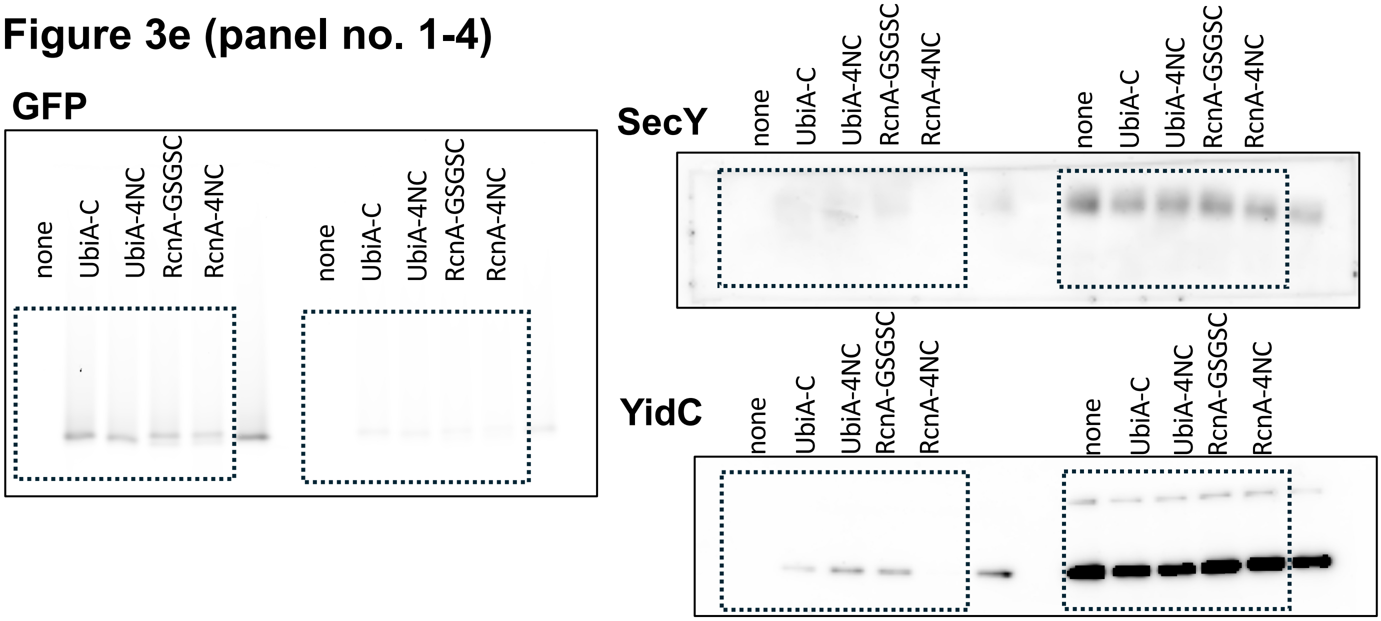

These panels are from the same gel

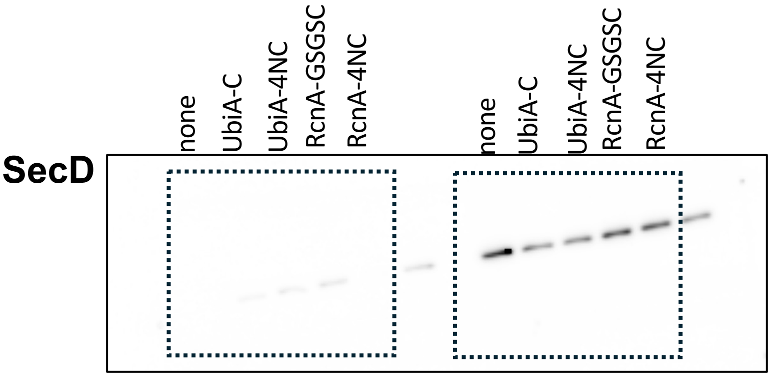

This panel is the same sample but different gel

Figure 3f lower panel

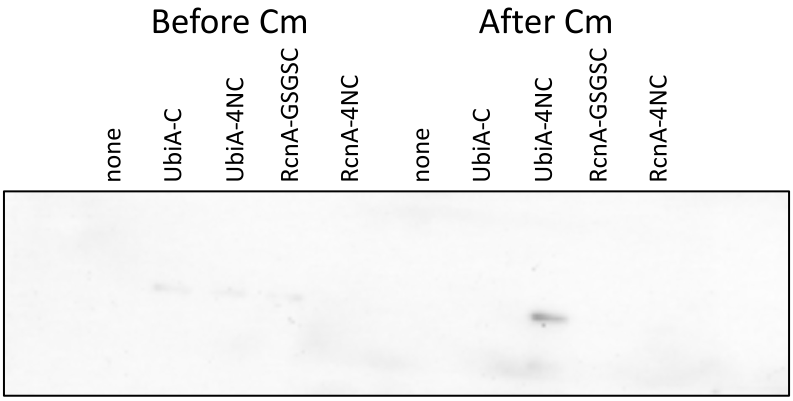

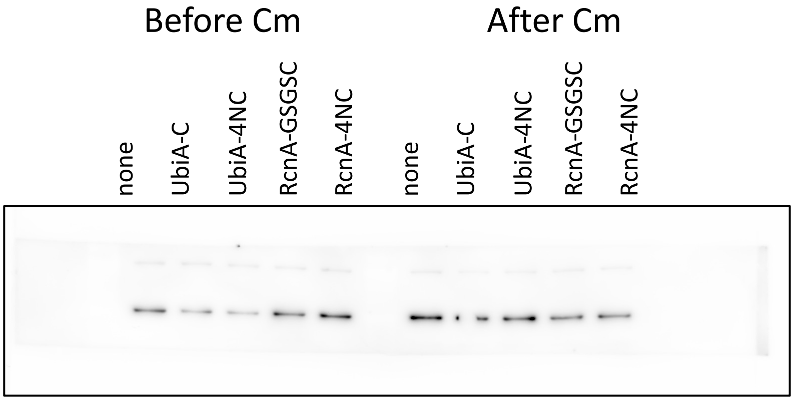

Supplement: Supplementary file 6 — Source Data [file 41467_2024_54575_MOESM6_ESM.zip › source data PDF/Fig. 3.pdf]
