# Supplementary figures and images for: Features of membrane protein sequence direct post-translational insertion
Source: Nat Commun. 2024 Nov 25;15:10198. doi: 10.1038/s41467-024-54575-6 (PMC11589881; doi:10.1038/s41467-024-54575-6)

**Fig. 4d.** Asterix (\*) Denotes unidentified bands in ATP6V0C Western blot.

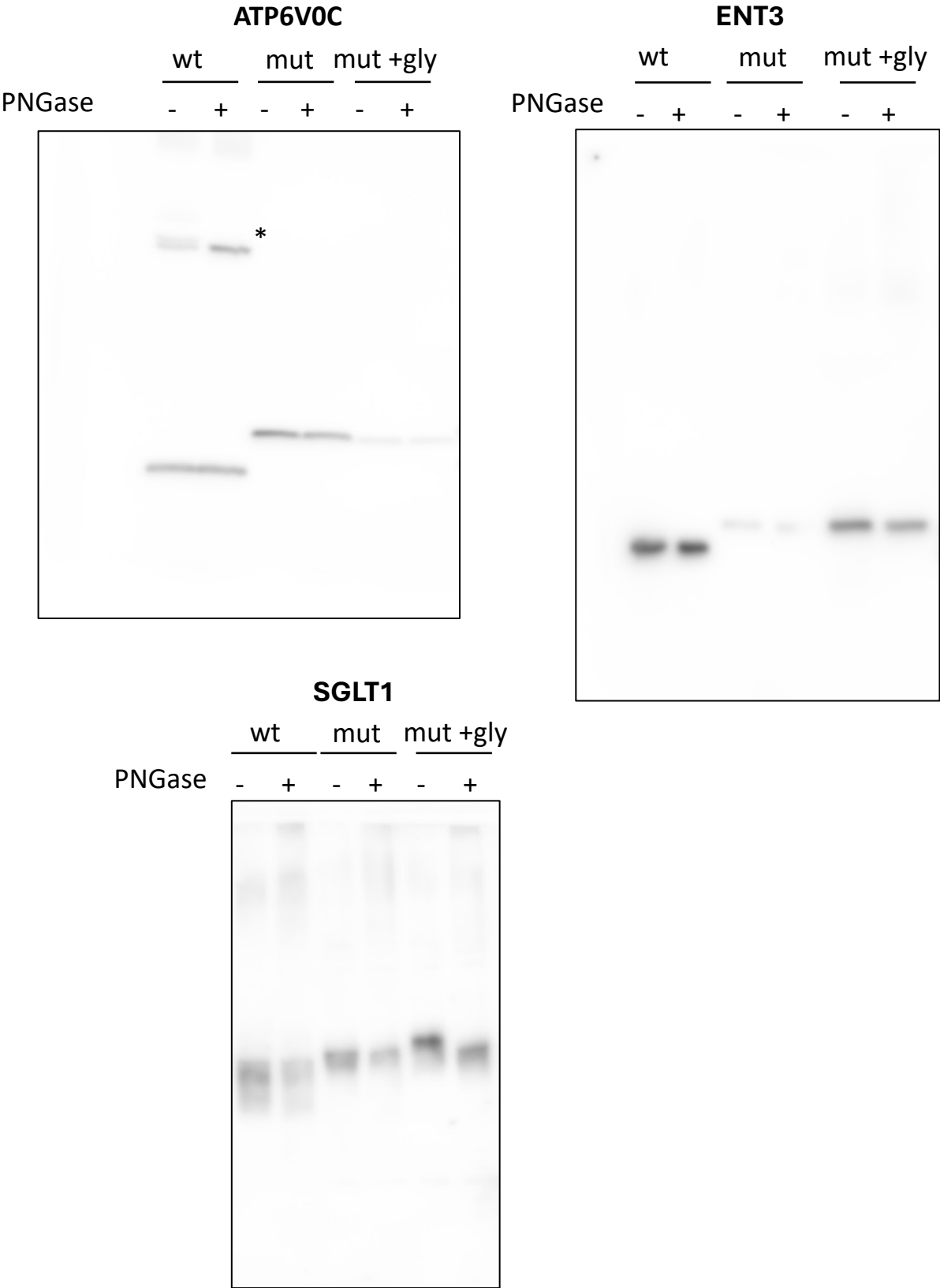

Supplement: Supplementary file 6 — Source Data [file 41467_2024_54575_MOESM6_ESM.zip › source data PDF/Fig. 4.pdf]

Supplementary Fig. 9

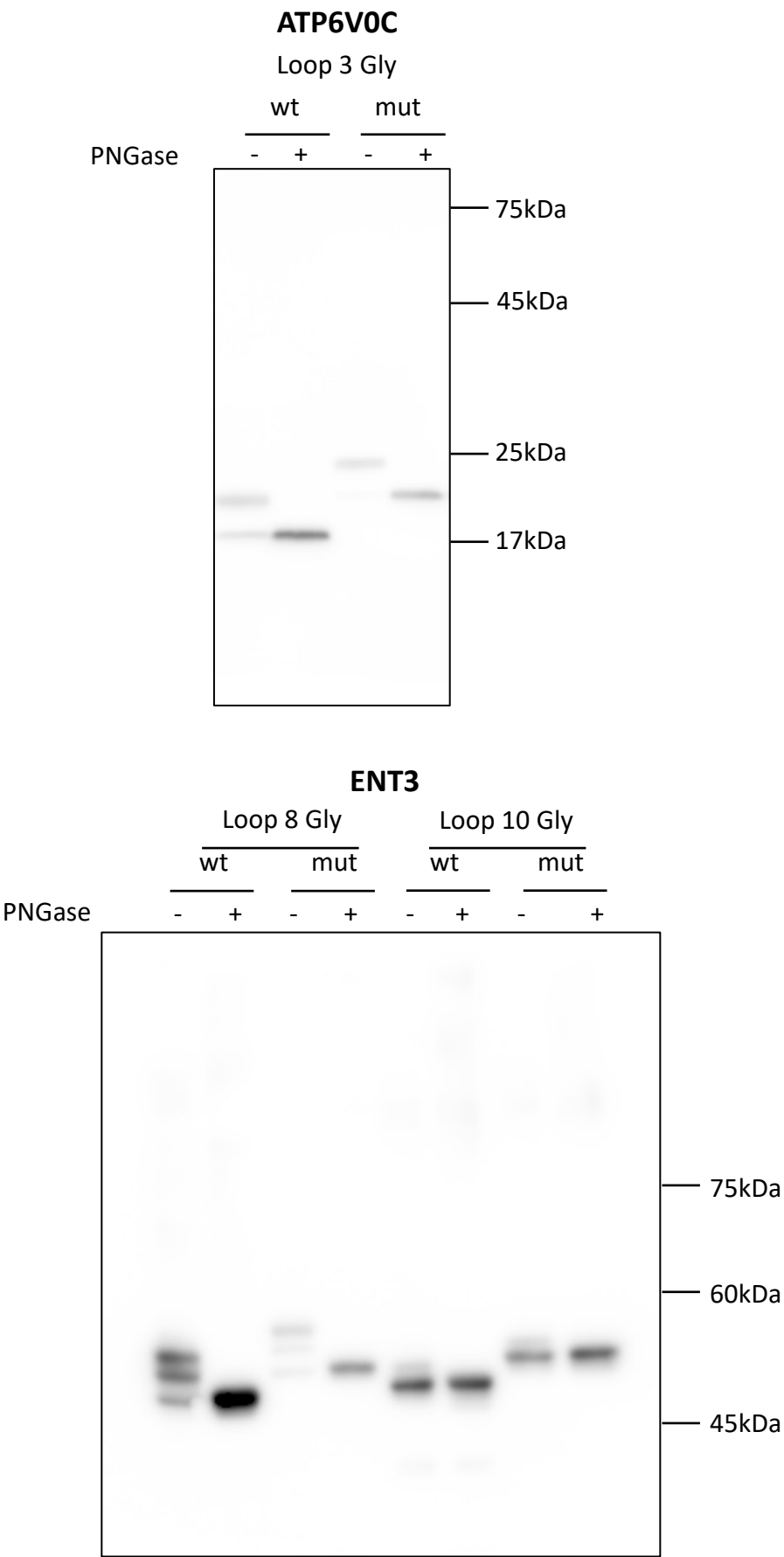

Supplement: Supplementary file 6 — Source Data [file 41467_2024_54575_MOESM6_ESM.zip › source data PDF/Supplementary Fig. 9.pdf]

Supplementary Figure 4a,b

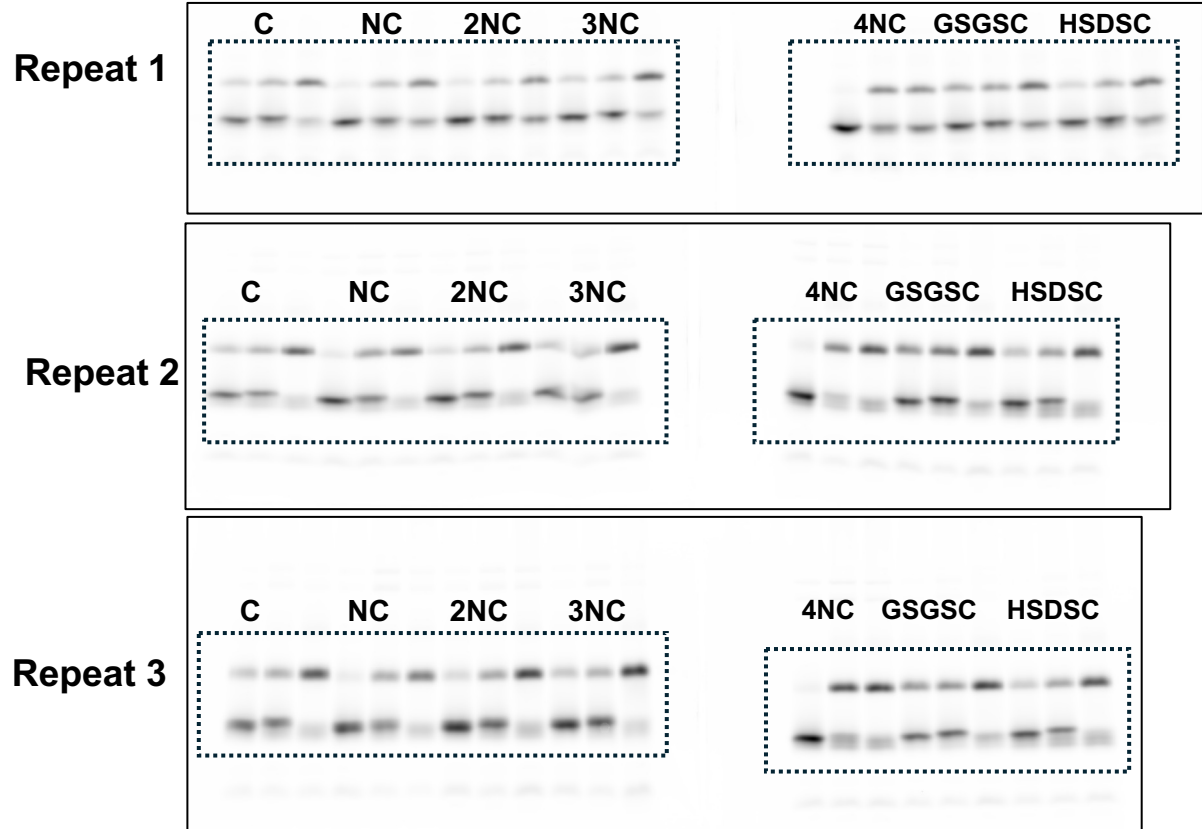

Supplementary Figure 4c,d

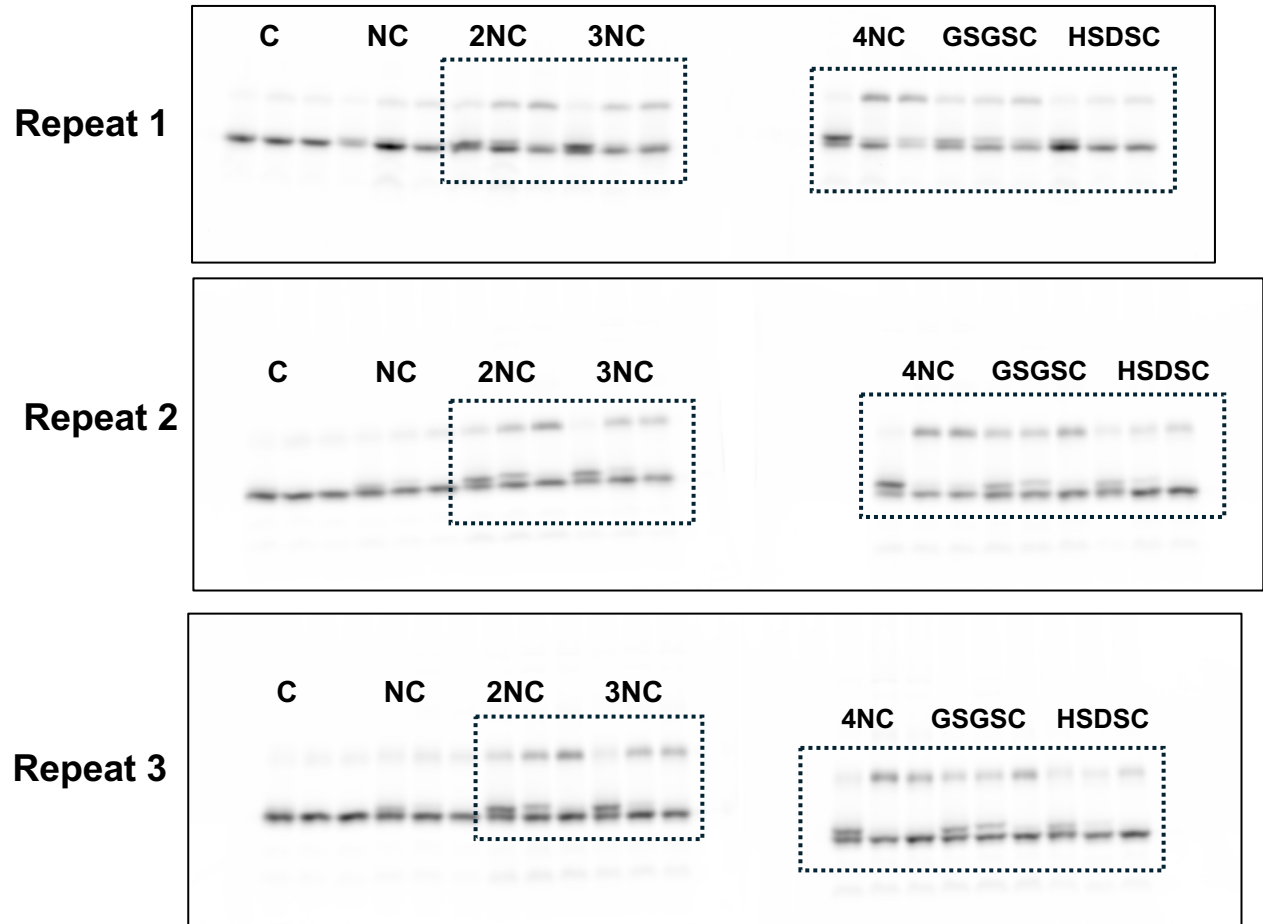

Supplement: Supplementary file 6 — Source Data [file 41467_2024_54575_MOESM6_ESM.zip › source data PDF/Supplementary Fig. 4.pdf]

Supplementary Figure 3

UbiA 291C

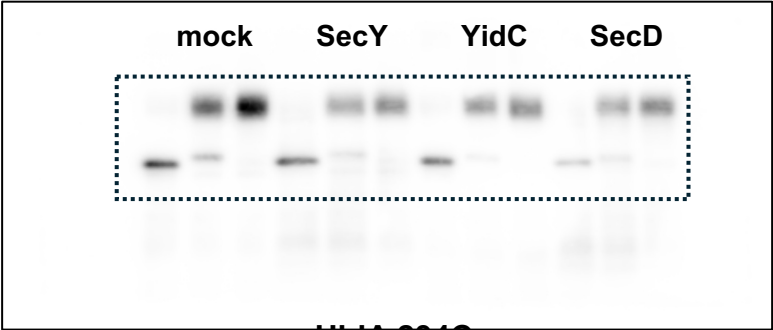

UbiA 234C

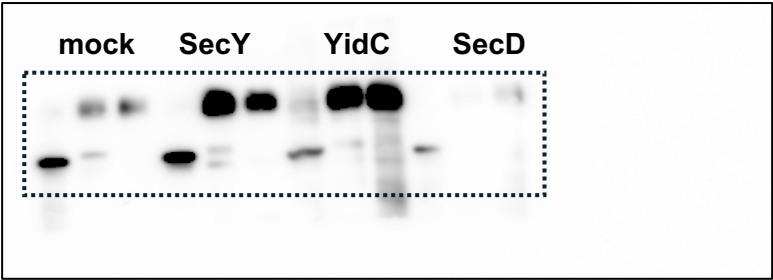

UbiA 291C

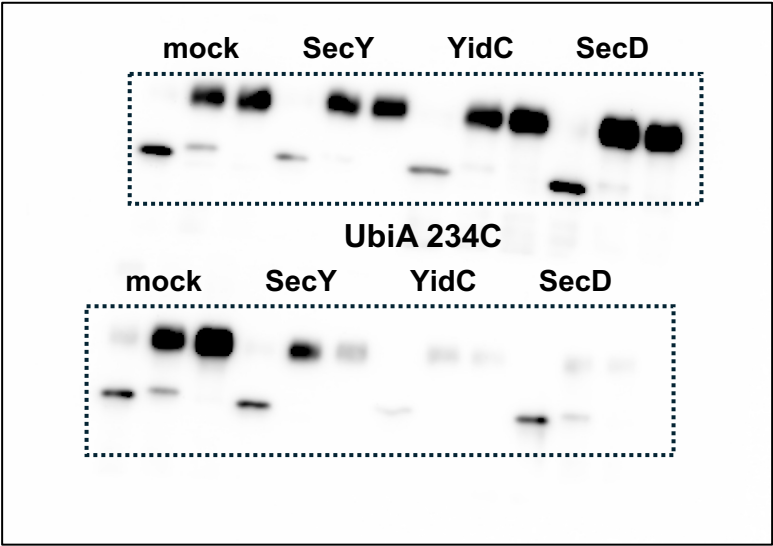

Supplement: Supplementary file 6 — Source Data [file 41467_2024_54575_MOESM6_ESM.zip › source data PDF/Supplementary Fig. 3.pdf]

**supplementary Fig. 8**

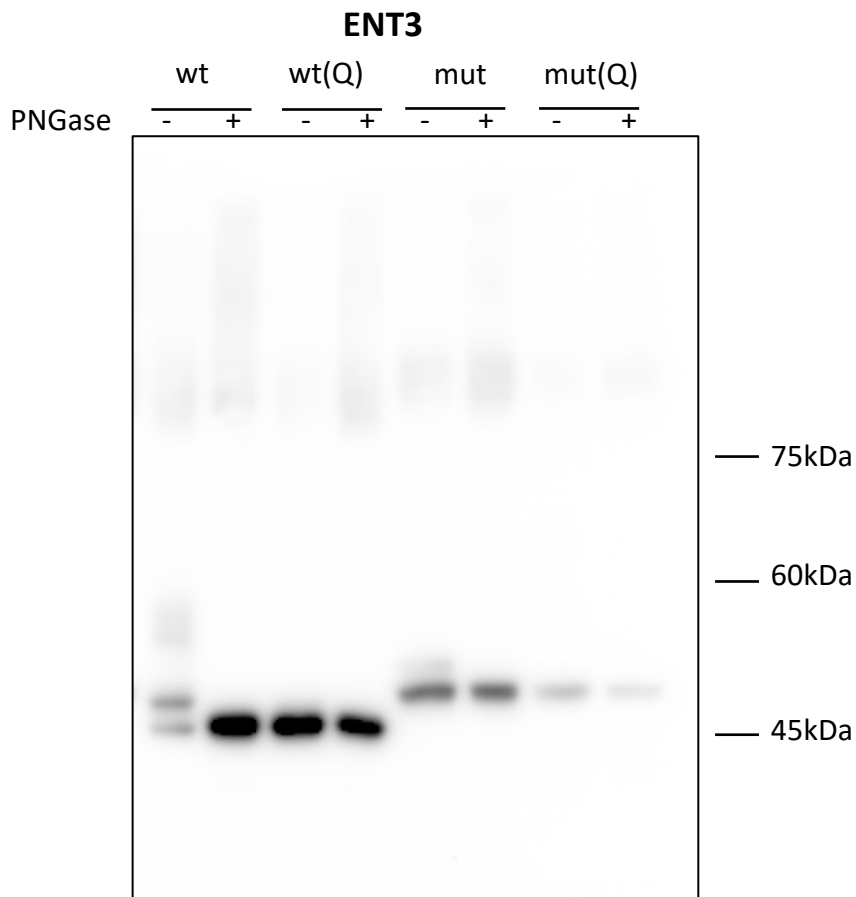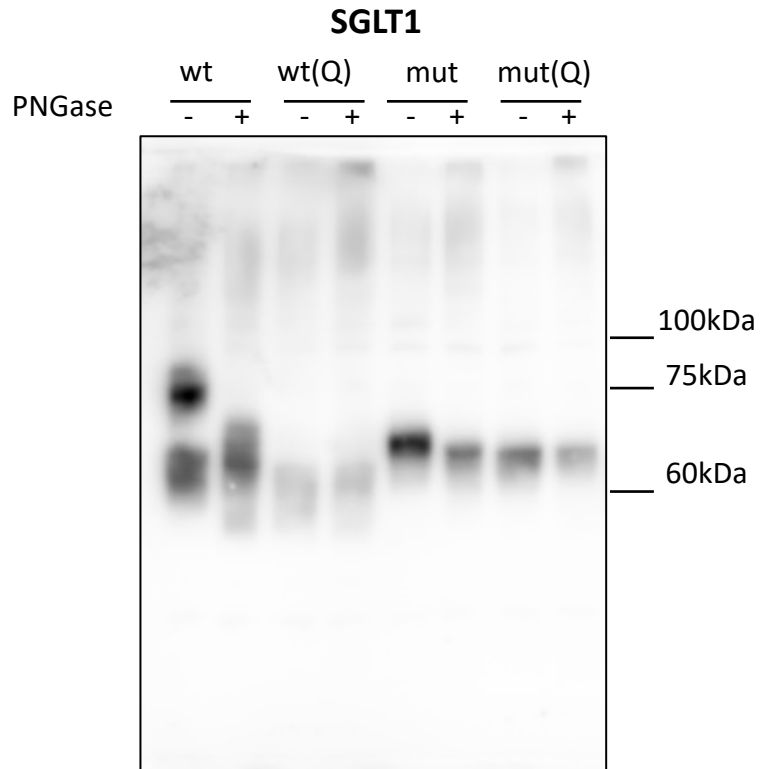

Supplement: Supplementary file 6 — Source Data [file 41467_2024_54575_MOESM6_ESM.zip › source data PDF/Supplementary Fig. 8.pdf]

Figure 2: UbiA

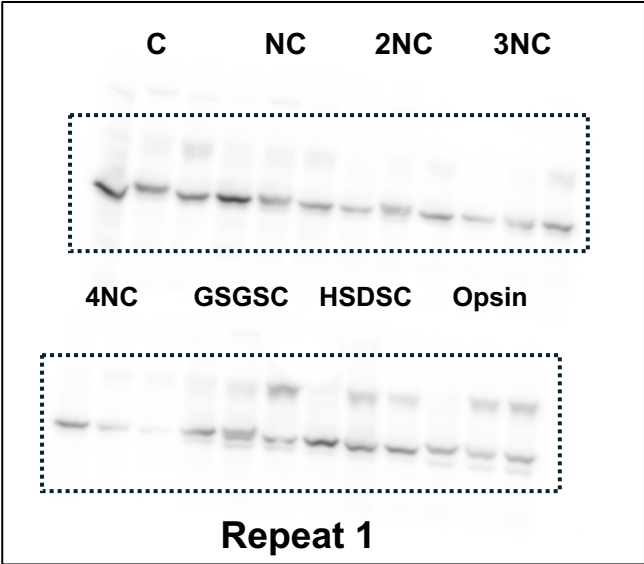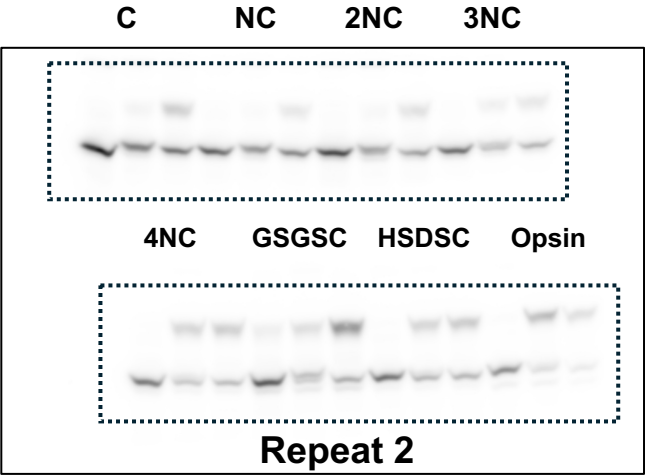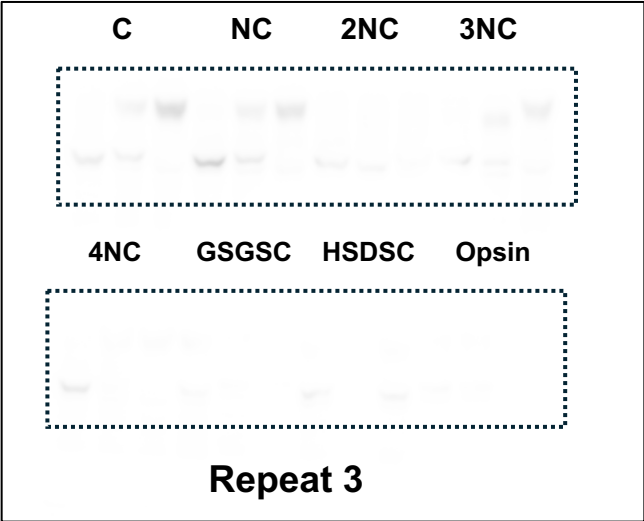

Figure 2: RcnA

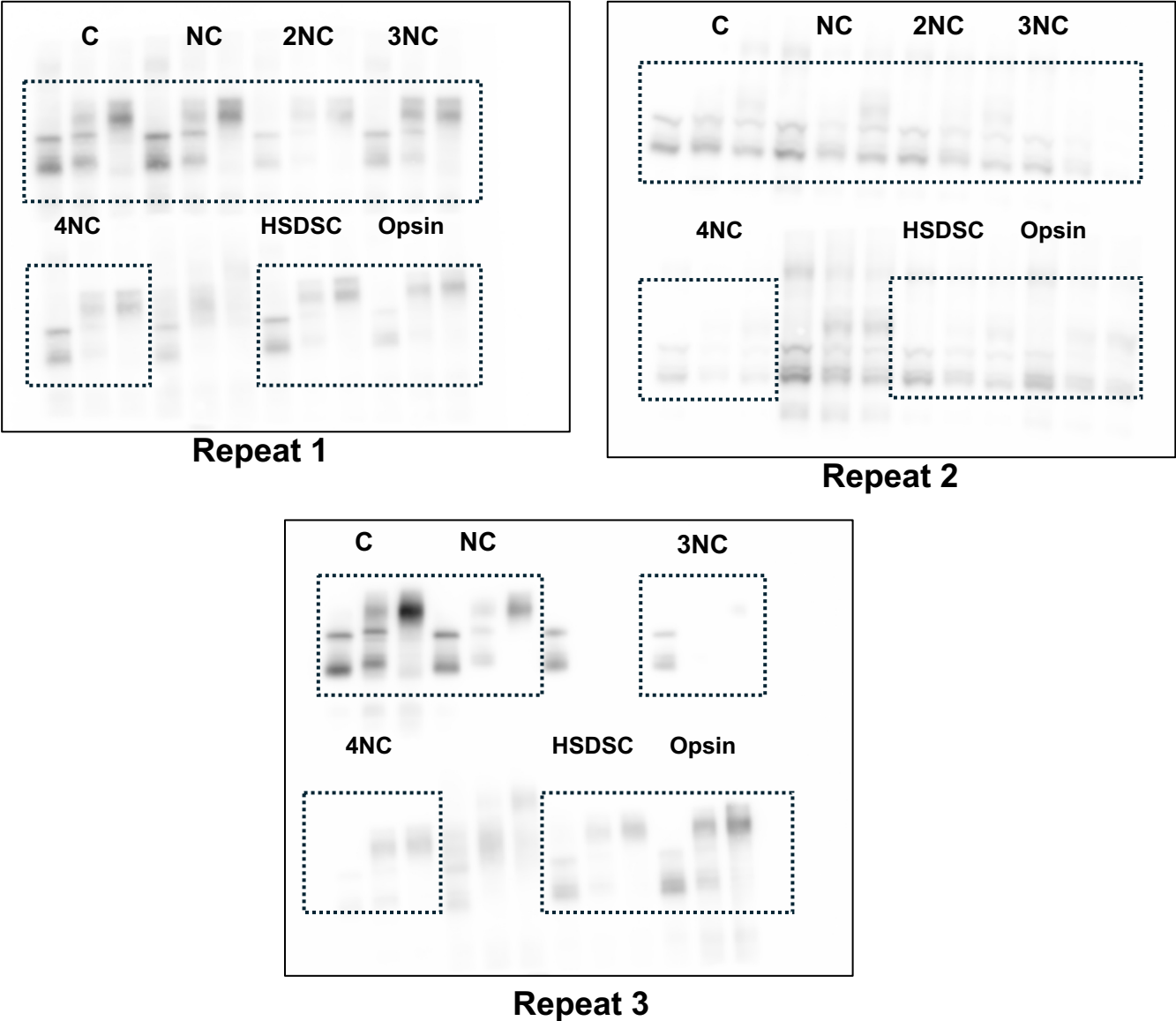

Figure 2: RcnA (GSGSC)

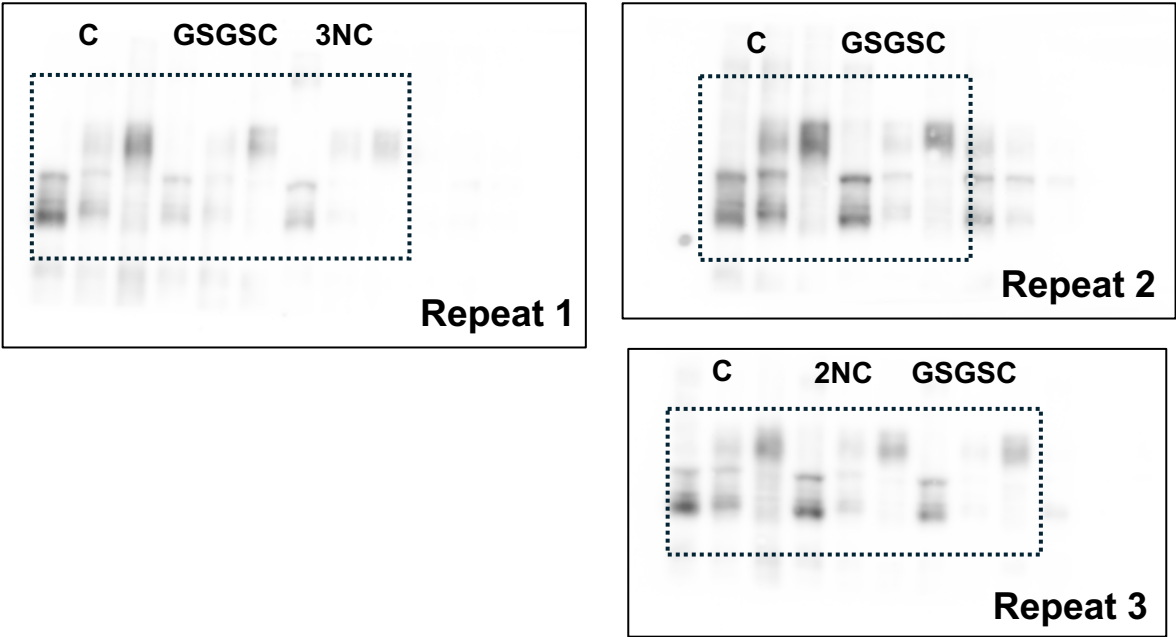

Supplement: Supplementary file 6 — Source Data [file 41467_2024_54575_MOESM6_ESM.zip › source data PDF/Fig. 2.pdf]
